# Dietary Choline-Enhanced Skin Immune Response of Juvenile Grass Carp Might Be Related to the STAT3 and NF-kB Signaling Pathway (*Ctenopharyngodon idella*)

**DOI:** 10.3389/fnut.2021.652767

**Published:** 2021-05-20

**Authors:** Ze-Hong Yuan, Lin Feng, Wei-dan Jiang, Pei Wu, Yang Liu, Jun Jiang, Sheng-yao Kuang, Ling Tang, Xiao-qiu Zhou

**Affiliations:** ^1^Animal Nutrition Institute, Sichuan Agricultural University, Chengdu, China; ^2^Fish Nutrition and Safety Production University Key Laboratory of Sichuan Province, Sichuan Agricultural University, Chengdu, China; ^3^Key Laboratory for Animal Disease-Resistance Nutrition of China Ministry of Education, Sichuan Agricultural University, Chengdu, China; ^4^Animal Nutrition Institute, Sichuan Academy of Animal Science, Chengdu, China

**Keywords:** juvenile grass carp (*Ctenopharyngodon idella*), immune function, choline, skin, JAK/STAT3, NF-κB signaling

## Abstract

To investigate the effects and potential mechanisms of dietary choline on immune function in the skin of juvenile grass carp (*Ctenopharyngodon idella*), fish were fed different diets containing different levels of choline (142. 2, 407.4, 821.6, 1215.8, 1589.3, and 1996.6 mg/kg) for 70 d and then sampled after a 6-d challenge test. The results exhibited that dietary choline (1) advanced the contents of phosphatidylcholine (PC), betaine, and choline in grass carp skin (*P* < 0.05) and upregulated the mRNA abundance of choline transporter high-affinity choline transporter 1 (CHT1), choline transporter-like protein 1 (CTL1), and choline transporter-like protein 5 (CTL5), indicating that dietary choline could increase the contents of choline which might be connected with choline transporters in the grass carp skin; (2) receded skin rot symptom after infection with *A. hydrophila* (Aeromonas hydrophila), increased the levels of IgM, C4, and C3 and the activities of acid phosphatase (ACP) and lysozyme (LZ), raised mucin2, β-defensin, hepcidin, and LEAP-2B mRNA abundance (rather than LEAP-2A), downregulated pro-inflammatory cytokine mRNA abundance (IFN-γ2, IL-15, TNF-α, IL-6, IL-12P40, and IL-1β) in skin of juvenile grass carp (*P* < 0.05), and upregulated anti-inflammatory cytokine mRNA abundance (IL-10, IL-4/13A, TGF-β1, IL-11, and IL-4/13B) in grass carp skin (*P* < 0.05), demonstrating that choline enhanced the skin immune function; and (3) downregulated the mRNA abundance of IKKγ, NF-κBp52, IKKβ, c-Rel, NF-κBp65, STAT3b2, STAT3b1, JAK1, and JAK2 as well as protein level of NF-κBp65, p-STAT3 Tyr705, and p-STAT3 Ser727 in nucleus and inhibited the mRNA and protein level of IkBα (*P* < 0.05), indicating that choline-enhanced immune function might be relevant to the JAK1, 2 /STAT3, and NF-κB signaling pathway in fish skin. In conclusion, choline enhanced the skin immune function which might be related to JAK1, 2/STAT3, and NF-κB signaling molecules in fish. Furthermore, based on immune indices of grass carp (9.28–108.97 g) skin (C3 and IgM contents as well as ACP activities), the choline requirements were estimated to be 1475.81, 1364.24, and 1574.37 mg/kg diet, respectively.

## Introduction

Choline is an indispensable vitamin B for fish (1). Our previous study found that optimal choline supplementation promoted the growth performances of grass carp (*Ctenopharyngodon idella*) (2, 3). Skin is a crucial immune organ in fish (4), whose health is important for fish growth and disease resistance (5). Nevertheless, no studies had reported the effect of choline on grass carp skin. Intensive aquaculture increases disease infection risk of fish (6). Improving immunity is crucial for the prevention and control of diseases in fish (7). Fish skin immune function is tightly correlated with specific immune factors like immunoglobulins and non-specific immune factors like LZ (2). Furthermore, fish skin immunity is closely related to the cytokines. NF-κB and STAT3 each control the expression of a large number of downstream genes that control cell proliferation, survival, stress responses, and immune functions. Some of the target genes for NF-κB and STAT3 overlap, and in addition, the two transcription factors are engaged in both positive and negative cross talk (8). Murray (9) found that anti-inflammatory cytokines could be regulated by STAT3 as well as upstream signaling molecule Janus kinases (JAK) in humans (9), while pro-inflammatory cytokine expression could be mediated by NF-κB (10). Choline enters the cell via choline transporters and produces corresponding metabolites to play biological functions (11). Betaine is one of vital metabolites which could decrease the content of tumor necrosis factor-α (TNF-α) in rat liver (12, 13). In mouse brain, choline enhanced the acetylcholine (ACh) level which could activate the JAK2/STAT3 signaling pathway in mouse PC12 cells (14, 15). In human, choline synthesis PC restrained the NF-κBp65 protein level in rat IEC-6 cells (16, 17). Thus, there might be a relationship between immune function and choline, which may be related to the JAK/STAT3 and NF-κB signaling pathway in fish skin.

Most researches of vitamins concentrated on liver health and nutritional requirements (1, 18). So far, reports regarding the impacts of vitamin on fish skin immunity are scattered. However, the following restrictions still remain: (1) in limited researches, we found that those researches are not deeply and systematically enough. Most studies mainly focused on the antibiotic substance contents and the inflammatory factor gene expression and did not investigate the involved mechanisms (19, 20). (2) The in-depth mechanisms of various vitamins on fish skin immune function are contrasting. For example, dietary VB7 supplement-enhanced skin immune function might be connected with key enzyme activity such as acid phosphatase (ACP) (20), whereas VC did not change the activity of ACP in fish skin (19). Hence, it is necessary to systematically research the impacts of choline on fish skin immunity.

In the current study, the growth trial was similar to our prior study. The study determines the dietary choline-enhanced fish growth performances (3) which were influenced by the skin immune function (21). Grass carp (*Ctenopharyngodon idella*) is a typical herbivorous finfish without stomach. In its natural environment, it eats water plant. Grass carp is the largest aquaculture of fish in China. The breeding of grass carp is of great significance to the whole fishery of China (22). Red skin disease is one of the main diseases threatening fish culture, which often occurs in grass carp. Hence, we explored the influences of choline on fish skin immune function, which partially declared the impact of choline on skin immune function and underlying mechanisms in fish. Simultaneously, vitamin requirements based on fish production performance are lower than the immune function of juvenile grass carp (19), so we determined the optimum choline requirements depending on the immune indicator for juvenile grass carp, which may provide the basis for production practice.

## Materials and Methods

### Experiments and Feeding Management

The current study used the same animal trial as our prior research (3). The feed formula is shown in Table 1, which was commensurate with prior research. Fishes were fed with six various gradient choline diets for 70 days. The choline levels were 142.2 (0), 407.4 (400), 821.6 (800), 1215.8 (1,200), 1589.3 (1,600), and 1996.6 (2,000) mg/kg in six diets, respectively (the value in front of the brackets is the measured value, and the value in the brackets is the design value). The actual choline level was determined by Ding and Mou (25).

**Table 1:** Composition and nutrients of basal diet.

**Ingredients**	**%**	**Nutrients**	**%**
Fish meal	3.97	Crude protein^d^	31.92
Casein	28.27	Crude lipid^d^	4.22
Gelatin	7.00	n-3 Fatty^e^	1.04
α-starch	24.00	n-6 Fatty^e^	0.96
Corn starch	18.72	Available phosphorus^f^	0.84
Fish oil	2.63		
Soybean oil	1.80		
Microcrystalline cellulose	5.00		
Ca(H_2_PO_4_)_2_	3.30		
Choline-free vitamin premix^a^	1.00		
Mineral premix^b^	2.00		
Choline chloride premix^c^	2.00		
DL-Met (99%)	0.26		
Ethoxyquin (30%)	0.05		

a *Per kilogram of choline-free vitamin premix (g kg^−1^): retinyl acetate (1,000,000 IU g^−1^), 0.400; cholecalciferol (500,000 IU g^−1^), 0.320; DL-a-tocopherol acetate (50%), 40.000; menadione (96%), 0.198; cyanocobalamin (1%), 0.940; D-biotin (2%), 0.750; folic acid (95%), 0.379; thiamine nitrate (98%), 0.133; ascorbyl acetate (95%), 4.737; niacin (99%), 2.576; meso-inositol (97%), 22.062; calcium-D-pantothenate (90%), 2.778; riboflavin (80%), 0.775; pyridoxine hydrochloride (98%), 0.115. All ingredients were diluted with cornstarch to 1 kg*.

b *Per kilogram of mineral premix (g kg^−1^): MnSO_4_·H_2_O (31.8% Mn), 3.098; MgSO_4_·H_2_O (15.0% Mg), 237.840; FeSO_4_·H_2_O (30.0% Fe), 15.000; ZnSO_4_·H_2_O (34.5% Zn), 7.860; CuSO_4_·5H_2_O (25.0% Cu), 0.600; CaI_2_ (3.2% I), 1.560; Na_2_SeO_3_ (44.7% Se), 0.132. All ingredients were diluted with corn starch to 1 kg*.

c *Per kilogram of choline chloride premix (g/kg): premix was added to obtain the graded level of choline. Each choline chloride mixture was diluted with cornstarch to 1 kg referenced to Wu et al. (23)*.

d *Crude protein and crude lipid contents were measured values*.

e *n-3 and n-6 were calculated by NRC (2011) contents referenced to Zeng et al. (24)*.

f *Available phosphorus were calculated according to NRC (2011)*.

All experimental procedures were approved by the Animal Protection Advisory Committee of Sichuan Agricultural University (23). Fishes were purchased from the fishery (Chengdu, China). Before the experiment, a 4-week feeding was adapted to the environment. After that, 1,440 fishes with average weight of 9.29 g were randomly assigned to 36 aquariums (0.144 m^3^). The same continuous aeration and recirculating water was maintained to each aquarium (26). During the growth experiment, the dissolved oxygen level ranged from 6.2 mg/L to 7.0 mg/L; pH was measured at 7.0 ± 0.3 and water temperature at 28 ± 2°C. Fishes were fed four times every day (23, 27).

### Challenge Test

The bacteria were supplied by the College of Veterinary Medicine, Sichuan Agricultural University. Prior to initiation of the challenge test, based on skin lesion morbidity and without causing any death, we determined an appropriate challenge concentration (2.1 × 10^6^ CFU/ml) after injection-graded levels (physiological saline, 2.1 × 10^5^, 2.1 × 10^6^, 2.1 × 10^7^, and 2.1 × 10^8^ CFU/ml) of Aeromonas hydrophila (*A. hydrophila*) in fish within 6 days (28).

After a 70-day growth experiment, we used a challenge test to investigate the effect of choline diets on fish immune function of juvenile grass carp. Forty-two fishes (same weight) from each treatment were intraperitoneal challenge injection with 2.1 × 10^6^ CFU/ml *A. hydrophila*. After injection for 6 days, the symptoms were recorded and the skin of each group was put into liquid nitrogen and reserved in −80°C.

### Measurement of Choline Metabolite Contents and Immune Parameter Activities

A preparation of 10% skin tissue homogenate was used to measure choline metabolite content and antibacterial material level as previously described (6). IgM, C4, C3 ACP, and LZ were determined using the corresponding kit (Nanjing Jiancheng Bioengineering Institute, Nanjing, China) following the manufacturer's protocols according to Takemura (29) and Huang et al. (30). LZ used turbidimetry; ACP used the colorimetric method; and IgM, C4, and C3 used immunoturbidimetry. The levels of ACh, betaine, PC, and choline were determined by ELISA kits (Shanghai Kexing Trading Co., Ltd, China) following the manufacturer's protocols and measured spectrophotometrically at a wavelength of 450 nm. The levels of ACh, betaine, PC, and choline were calculated on the basis of standard curves.

### Quantitative Real-Time PCR

The skin total RNA was extracted using the RNAiso Plus kit (TaKaRa Bio Inc., Japan) following the specification. Thenceforth, the instruction was referred to synthase cDNA using the PrimeScript™ RT Reagent Kit (TaKaRa). The qRT-PCR primers were referred to the published sequences of grass carp in our lab prior to the study, which are shown in Table 2. The gene amplification efficiency was measured by the melting curve. The 2^−ΔΔCT^ method was used to calculate the gene expression as described by Yao et al. (31) and Zhang et al. (32).

**Table 2:** Real-time PCR primer sequences.

**Target gene**	**Primer sequence forward (5′ → 3′)**	**Primer sequence reverse (5′ → 3′)**	**Temperature (°C)**	**Accession number**
IL-6	CAGCAGAATGGGGGAGTTATC	CTCGCAGAGTCTTGACATCCTT	62.3	KC535507.1
IL-12p35	TGGAAAAGGAGGGGAAGATG	AGACGGACGCTGTGTGAGTGTA	55.4	KF944667.1
IL-12p40	ACAAAGATGAAAAACTGGAGGC	GTGTGTGGTTTAGGTAGGAGCC	59	KF944668.1
IL-15	CCTTCCAACAATCTCGCTTC	AACACATCTTCCAGTTCTCCTT	61.4	KT445872.1
IL-17D	GTGTCCAGGAGAGCACCAAG	GCGAGAGGCTGAGGAAGTT T	62.3	KF245426.1
IL-4/13A	CTACTGCTCGCTTTCGCTGT	CCCAGTTTTCAGTTCTCTCAGG	55.9	KT445871.1
IL-4/13B	TGTGAACCAGACCCTACATAACC	TTCAGGACCTTTGCTGCTTG	55.9	KT625600.1
TNF-α	CGCTGCTGTCTGCTTCAC	CCTGGTCCTGGTTCACTC	58.4	HQ696609
IFN-γ2	TGTTTGATGACTTTGGGATG	TCAGGACCCGCAGGAAGAC	60.4	JX657682
IL-1β	AGAGTTTGGTGAAGAAGAGG	TTATTGTGGTTACGCTGGA	57.1	JQ692172
TGF-β1	TTGGGACTTGTGCTCTAT	AGTTCTGCTGGGATGTTT	55.9	EU099588
TGF-β2	TACATTGACAGCAAGGTGGTG	TCTTGTTGGGGATGATGTAGTT	55.9	KM279716
IL-10	AATCCCTTTGATTTTGCC	GTGCCTTATCCTACAGTATGTG	61.4	HQ388294
IL-11	GGTTCAAGTCTCTTCCAGCGAT	TGCGTGTTATTTTGTTCAGCCA	57	KT445870.1
Hepcidin	AGCAGGAGCAGGATGAGC	GCCAGGGGATTTGTTTGT	59.3	JQ246442.1
LEAP-2A	TGCCTACTGCCAGAACCA	AATCGGTTGGCTGTAGGA	59.3	FJ390414
LEAP-2B	TGTGCCATTAGCGACTTCTGAG	ATGATTCGCCACAAAGGGG	59.3	KT625603.1
βdefensin-1	TTGCTTGTCCTTGCCGTCT	AATCCTTTGCCACAGCCTAA	58.4	KT445868.1
Mucin2	GAGTTCCCAACCCAACACAT	AAAGGTCTACACAATCTGCCC	60.4	KT625602
CTL1	GAACCGCAGGAAGTCCAGTG	GCTGACAGGCGAGGATGAACT	60.7	MN904650
CTL2	AACTTCGTGACAGCATTGGG	ATGGCAAGAATGAGGGAACC	58.6	MN904651
CTL4	GGTCATTGCGATGGTGGTC	CAGATACCGAAGGCTCCGAC	59.2	MN904652
CTL5	GCAAAGGAAATCGGCATC	GCGGTGAACCTCAGCAGC	57.8	MN904653
CHT1	TCCTCATCACCCACACGA	CCGACTCCTCCATCCTCTC	55.4	MN904654
NF-κB p52	TCAGTGTAACGACAACGGGAT	ATACTTCAGCCACACCTCTCTTAG	58.4	KM79720
NF-κB p65	GAAGAAGGATGTGGGAGATG	TGTTGTCGTAGATGGGCTGAG	62.3	KJ526214
c-Rel	GCGTCTATGCTTCCAGATTTACC	ACTGCCACTGTTCTTGTTCACC	59.3	KT445865
IκBα	TCTTGCCATTATTCACGAGG	TGTTACCACAGTCATCCACCA	62.3	KJ125069
IKKα	GGCTACGCCAAAGACCTG	CGGACCTCGCCATTCATA	60.3	KM279718
IKKβ	GTGGCGGTGGATTATTGG	GCACGGGTTGCCAGTTTG	60.3	KP125491
IKKγ	AGAGGCTCGTCATAGTGG	CTGTGATTGGCTTGCTTT	58.4	KM079079
JAK1	TTTGCTGCACTGGTGGACA	GCGCAGGACATAGGTTCCTT	60.0	KT724352.1
JAK2	AGAGGCCATCGAGAGCTACT	TCATACGCCCCAACTGCAA	59.7	JF825474.1
JAK3	GCCGTTCAAGTGTCTGGAGA	AACTCAGCCTCCATGCACT	59.5	KU200686.1
TYK2	TTCGCCGTGTGTTTGCAAA	ACGCCAAAATGAGGAGCCA	59.7	KT724353.1
STAT3a	ACATTCCTGCTGCGCTTCA	ACGAGGATGTTGGTGGCAT	59.8	KC978890
STAT3b1	TCAACATGGCCCAGTGGAA	AGCGTTGCGTGAGATTCCT	59.4	KU559609
STAT3b2	GCTGACCAACCATCCAAA	CGGAGTAGTTTACACACGGAC	54.5	KU559610
β-Actin	GGCTGTGCTGTCCCTGTA	GGGCATAACCCTCGTAGAT	61.4	M25013

### Western Blotting

Lysis buffer, protease inhibitor cocktail, and phosphatase inhibitor cocktail were used to prepare skin homogenate (10). The protein level was determined by BCA assay kit (Beyotime Biotechnology Inc.). β-Actin, laminB1, p-STAT3Tyr705, NF-κBp65, and IκBα antibodies are similar to our early research (28, 33, 34). P-STAT3Ser727 antibodies were purchased from commercial antibodies (Hua'an Biology Co., Ltd.). LaminB1 and β-actin were used as control proteins for nuclei and cell total proteins, respectively. Equal amounts of protein were separated by sodium dodecyl sulfate polyacrylamide gel electrophoresis (SDS-PAGE) and transferred to a PVDF membrane. Membranes were blocked for 1 h at room temperature (RT) before being washed thrice with TBST (10 min each) and incubated with a primary antibody overnight at 4°C. Next, the membranes were again washed three times before incubation with an HRP-conjugated secondary antibody in TBST for 2 h. Immune complexes were visualized with an ECL kit (Millipore). Densitometric analyses of the protein bands were performed in ImageJ (NIH, USA). Different treatments were expressed relative to the level observed in the control group. The experiment was repeated at least three times, and similar results were obtained each time.

### Statistical Analysis

The Shapiro–Wilk and Levene tests were used to test the homogeneity and normal distribution of variance by SAS 8.1 (SAS Institute), respectively. One-way variance (ANOVA) was used to analyze the data (35). The significant differences between every treatment means were contrasted by Duncan's multiple-range tests (significant difference at the 5% level of significance, *P* < 0.05) (36). The choline requirement and correlations were estimated by broken-line mode and Pearson's correlation, respectively (23).

## Results

### The Contents of Choline and Its Metabolites and the Gene Expression of the Choline Transporter

**Figure 1:**
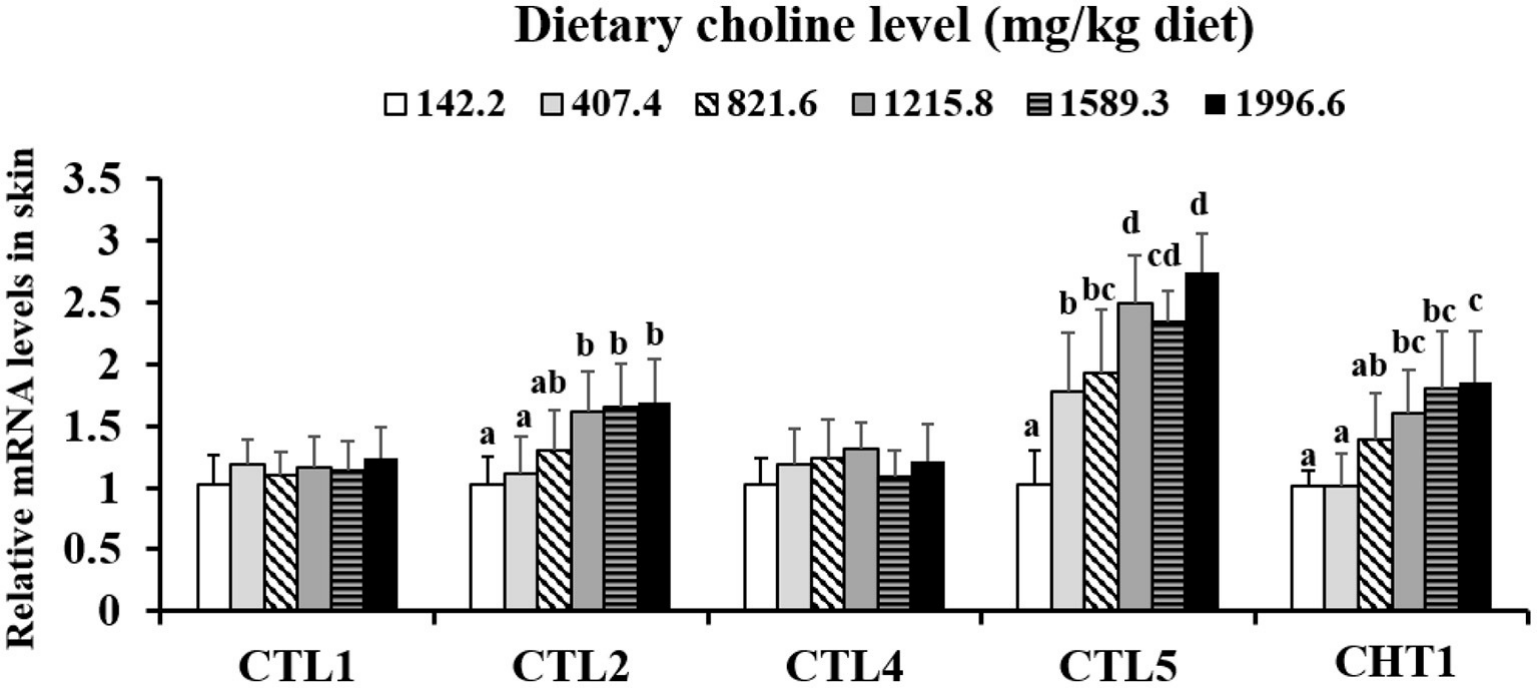
Effects of dietary choline on choline transporter gene level in the skin of juvenile grass carp (*Ctenopharyngodon idella*) after infection with *A*. *hydrophila*. Data represent means of six fish in each group, error bars indicate S.D. Values having different letters are significantly different (*P* < 0.05).

As shown in Table 3, the levels of PC, betaine, and choline were increased with dietary choline up to 1589.3, 1589.3, and 1215.8 mg/kg (*P* < 0.05), respectively, and then plateaued. However, the content of ACh did not change with the increase in choline content (*P* > 0.05). The choline transporter mRNA abundance of skin is exhibited in Figure 1; CTL2 and CTL5 mRNA abundance was raised with dietary choline by 1215.8 and 1589.3 mg/kg (*P* < 0.05), respectively, then plateaued. CHT1 mRNA abundance was slowly increased with dietary choline addition (*P* < 0.05). Dietary choline did not change the mRNA abundance of CTL1 and CTL4 in fish skin (*P* > 0.05).

**Table 3:** Effects of dietary choline (mg/kg diet) on choline, phosphatidylcholine (PC), betaine, and acetylcholine (ACh) contents in the skin of grass carp (*Ctenopharyngodon idella*)^1^.

	**Dietary choline levels (mg/kg diet)**					
**μg/g**	**142.2**	**407.4**	**821.6**	**1215.8**	**1589.3**	**1996.6**
Choline	165.73 ± 5.79^a^	169.51 ± 6.50^a^	172.10 ± 5.86^ab^	180.91 ± 4.12^c^	178.62 ± 5.75^bc^	180.13 ± 5.20^c^
Betaine	160.66 ± 10.49^a^	184.97 ± 17.15^b^	204.06 ± 5.46^c^	215.35 ± 10.03^cd^	230.80 ± 18.37^d^	223.33 ± 17.65^d^
ACh	81.67 ± 4.69	88.29 ± 4.13	88.45 ± 8.61	87.16 ± 6.48	86.68 ± 5.77	83.29 ± 6.68
PC	1208.99 ± 151.88^a^	1547.81 ± 136.72^b^	1592.76 ± 77.37^bc^	1721.05 ± 60.15^cd^	1740.24 ± 92.68^d^	1693.64 ± 95.1^cd^

1 *Values are means ± SD (n = 6), and different superscripts in the same row are significantly different (P < 0.05)*.

### Skin Rot Morbidity and Activities of Immune Parameters in Grass Carp Skin

**Figure 2:**
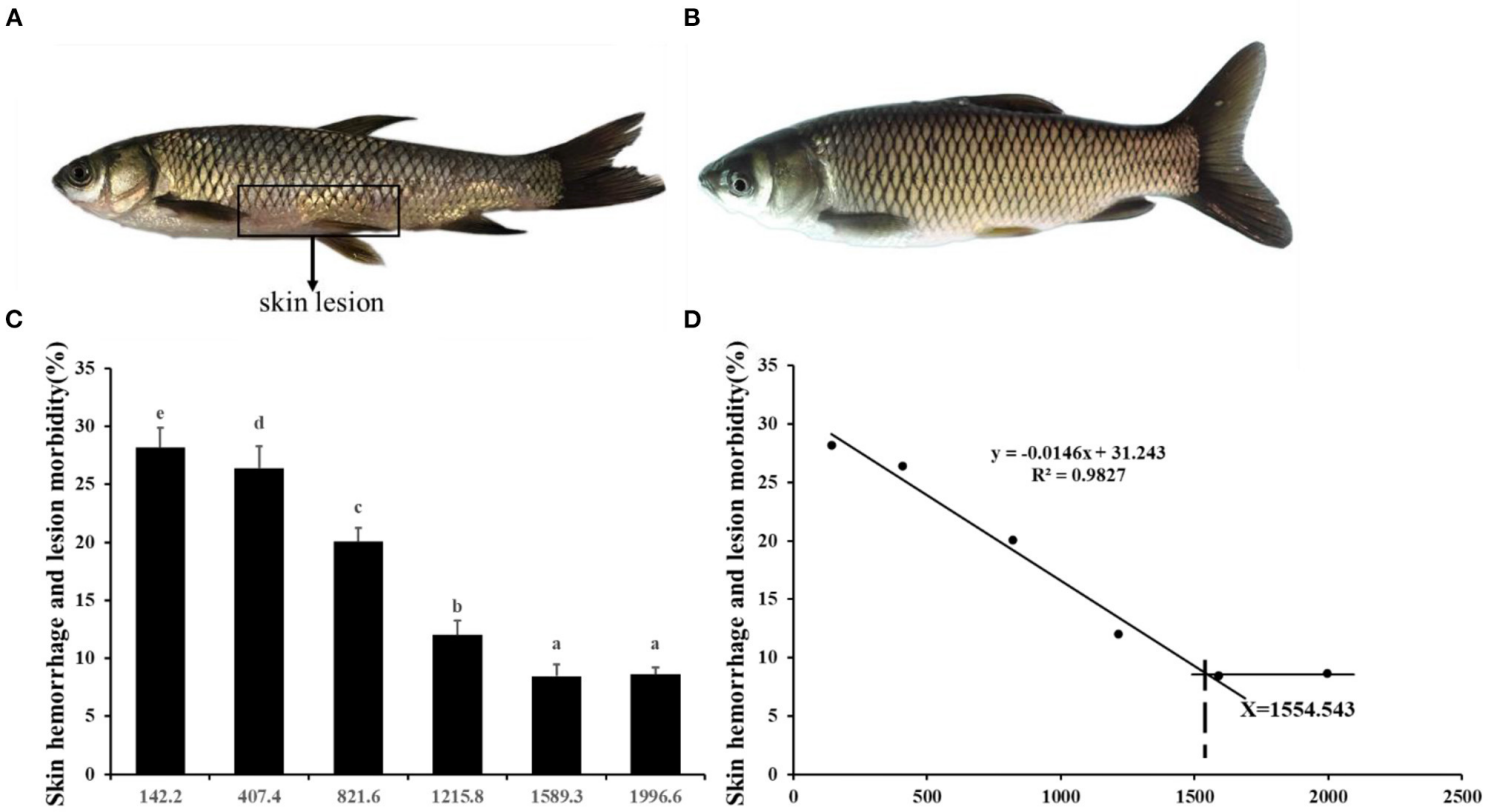
Effects of dietary choline level (mg/kg diet) on skin rot morbidity of juvenile grass carp (*Ctenopharyngodon idella*) after infection with *A. hydrophila*. **(A)** 142.2 mg/kg diet. **(B)** 1589.3 mg/kg diet. Values are means, and standard error of the mean represented by vertical bars. *N* = 6*5 for each choline level. Values having different letters are significantly different (*P* < 0.05). **(C)** Skin hemorrhage and lesion morbidity (%). **(D)** Choline requirement is assessed by morbidity.

**Figure 3:**
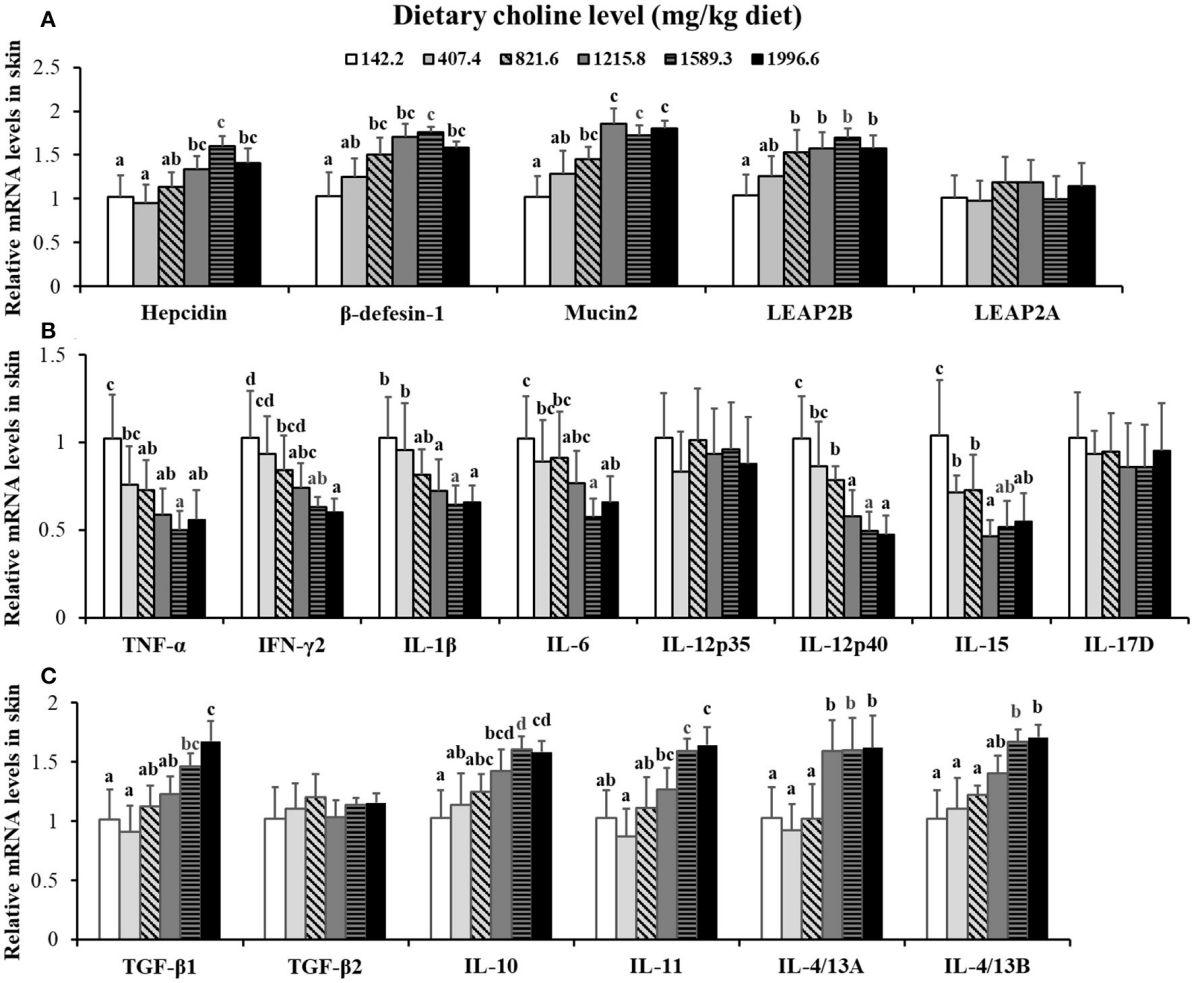
Effects of dietary choline on parameter mRNA levels in the skin of grass carp after infection with *A*. *hydrophila*. This analysis was repeated six times with similar results. Data represent means of six fish in each group, and error bars indicate S.D. Values having different letters are significantly different (*P* < 0.05). **(A)** Relative mRNA level of Hepcidin, β-defensin-1, Mucin2, LEAP-2A, and LEAP-2B in the skin. **(B)** Relative mRNA level of pro-inflammatory cytokines in the skin. **(C)** Relative mRNA levels of anti-inflammatory cytokines in the skin.

Choline insufficiency caused skin rot symptom, as shown in Figure 2. As exhibited in Table 4, the activities of ACP and LZ in grass carp skin increased as the dietary choline level increased to 1215.8 mg/kg (*P* < 0.05), then plateaued. The levels of C3, IgM, and C4 in the skin increased as dietary choline supplements to 1589.3, 821.6, and 1215.8 mg/kg (*P* < 0.05), respectively, then all plateaued. The antibacterial peptide mRNA abundance of the skin is presented in Figure 3A; mucin2 and LEAP-2B mRNA abundance was raised with dietary choline level addition to 1215.8 and 821.6 mg/kg (*P* < 0.05), respectively, then plateaued. The mRNA levels of hepcidin and β-defensin-1 increased with choline supplements to 1589.3 mg/kg (*P* < 0.05), then decreased. Nevertheless, mRNA abundance of LEAP-2A was not influenced by choline (*P* > 0.05). As exhibited in Figure 3B, the mRNA abundance of TNF-α, IL-15, IL-6, IL-12p40, and IL-1β in skin of juvenile grass carp decreased with dietary choline level increase to 821.6, 1215.8, 1589.3, 1215.8, and 1215.8 mg/kg, respectively (*P* < 0.05), and then plateaued. The mRNA abundance of IFN-γ2 in skin of juvenile grass carp decreased with dietary choline addition (*P* < 0.05). In Figure 3C, the mRNA abundance of anti-inflammatory cytokine IL-4/13B, IL-10, IL-11, and IL-4/13A in skin of fish (*P* < 0.05) was elevated with choline level increase to 1589.3, 1215.8, 1589.3, and 1589.3 mg/kg, respectively (*P* < 0.05). TGF-β1 mRNA abundance in fish skin was elevated with dietary choline addition (*P* < 0.05). Remarkably, the mRNA abundance of IL-17D, TGF-β2, and IL-12p35 was not impacted by dietary choline (*P* > 0.05).

**Table 4:** Effects of dietary choline (mg/kg diet) on immune parameters in juvenile grass carp (*Ctenopharyngodon idella*) skin^1^.

	**Dietary choline levels (mg/kg diet)**					
	**142.2**	**407.4**	**821.6**	**1215.8**	**1589.3**	**1996.6**
C3	11.08 ± 1.00^a^	15.25 ± 1.29^b^	16.62 ± 1.63^b^	19.24 ± 0.91^c^	20.97 ± 1.06^d^	20.33 ± 1.75^cd^
C4	1.20 ± 0.12^a^	1.98 ± 0.14^b^	3.07 ± 0.24^c^	4.07 ± 0.30^d^	3.51 ± 0.32^d^	3.40 ± 0.31^d^
IgM	31.60 ± 3.05^a^	30.53 ± 2.97^a^	40.94 ± 3.95^b^	42.02 ± 2.53^b^	43.13 ± 2.38^b^	41.79 ± 4.18^b^
ACP	66.83 ± 7.15^a^	87.72 ± 8.36^b^	88.47 ± 7.70^b^	101.58 ± 11.17^c^	117.07 ± 7.53^c^	111.39 ± 9.66^c^
LZ	81.01 ± 8.34^a^	107.51 ± 9.83^b^	114.73 ± 10.49^b^	136.99 ± 14.68^c^	139.07 ± 15.07^c^	128.97 ± 6.95^c^

1 *Values are means ± SD (n = 6), and different superscripts in the same row are significantly different (P < 0.05). Lysozyme activity (U/mg protein); ACP, acid phosphatase (U/mg protein); C3, complement 3 (mg/g protein); C4, complement 4 (mg/g protein); IgM, immunoglobulin M (mg/g protein)*.

### JAK/STAT3 Signaling Pathway and NK-kB Signaling Pathway mRNA Abundance in Fish Skin

**Figure 4:**
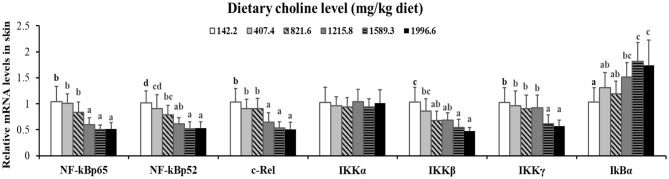
Effects of dietary choline level (mg/kg diet) on relative expression of NF-κB signaling molecules in skin of juvenile grass carp (*Ctenopharyngodon idella*) after infection with *A*. *hydrophila*. Data represent means of six fish in each group; error bars indicate S.D. Values having different letters are significantly different (*P* < 0.05).

**Figure 5:**
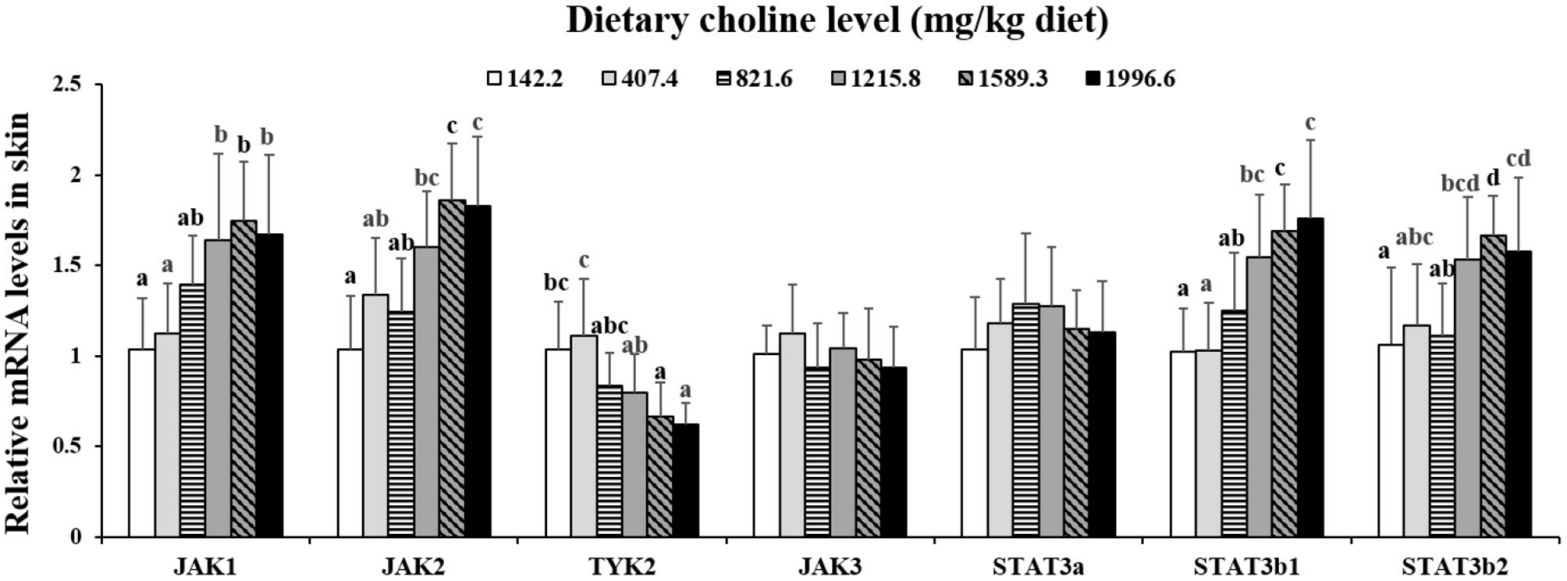
Effects of dietary choline level (mg/kg diet) on relative expression of JAK/STAT3 in skin of juvenile grass carp (*Ctenopharyngodon idella*) after infection with *A. hydrophila*. Data represent means of six fish in each group, error bars indicate S.D. Values having different letters are significantly different (*P* < 0.05).

**Figure 6:**
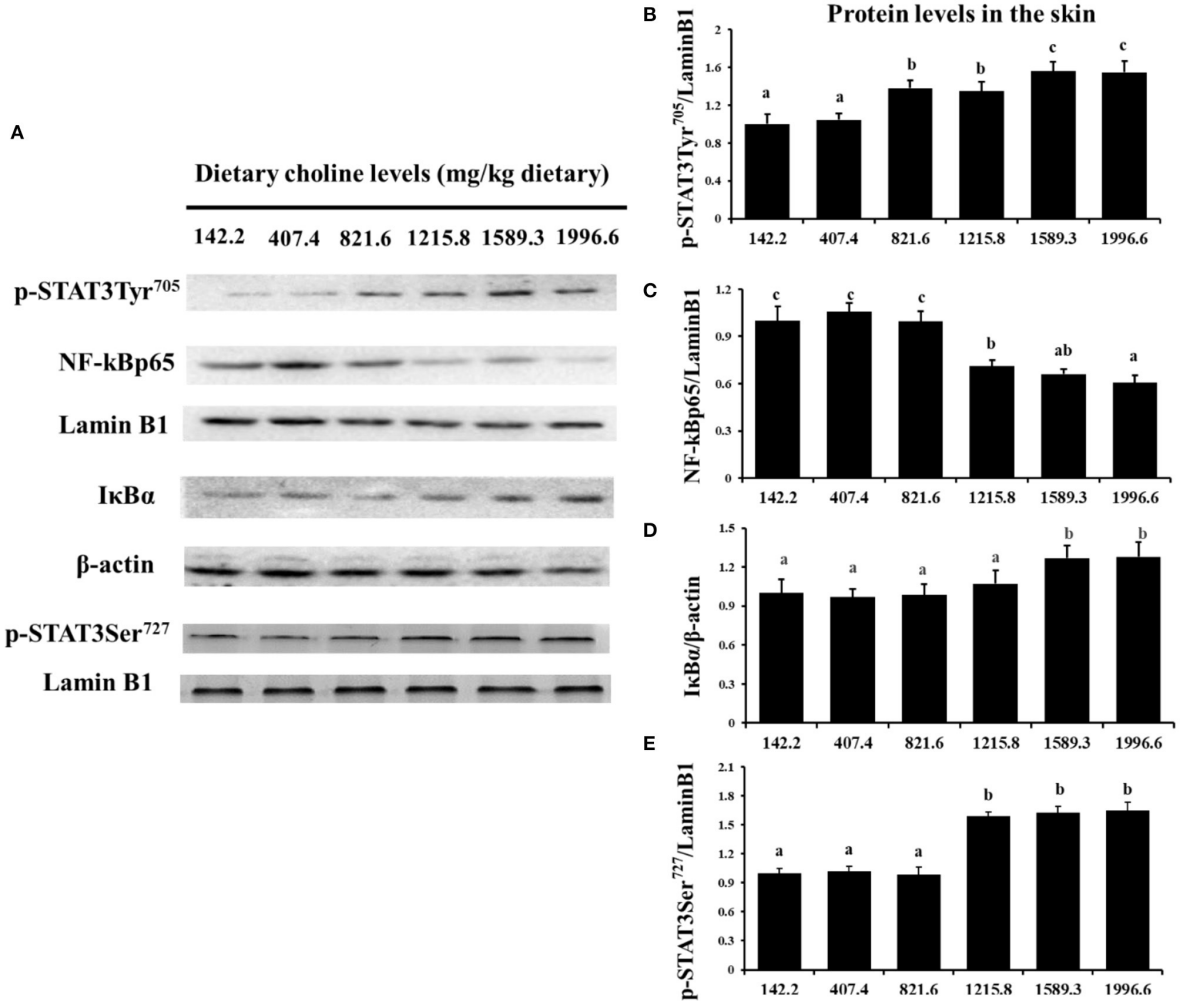
Western blot analysis of p-STAT3Tyr705, p-STAT3 Ser727, NF-κBp65, and IκBα protein level in the skin of juvenile grass carp fed diet containing different levels of choline after infection with *A. hydrophila*. Values are means (six replicates per group), and standard error represented by vertical bars. ^a,b,c^Mean values with unlike letters were significantly different between treatments (*P* < 0.05). **(A)** Protein bands of p-STAT3 Tyr705, NF-κBp65, and IκBα. **(B)** The relative protein expression of p-STAT3 Tyr705/laminB1. **(C)** The relative protein expression of NF-Kp65/laminB1. **(D)** The relative protein expression of IκBα/laminB1. **(E)** The relative protein expression of p-STAT3 Ser727/laminB1.

As exhibited in Figure 4, in juvenile grass carp skin, the mRNA abundance of IKK-γ, NF-κB p65, c-Rel, NF-κB p52, and IKK-β decreased with dietary choline levels of 1589.3, 1215.8, 1215.8, 1589.3, and 1589.3 mg/kg, respectively (*P* < 0.05), and then plateaued. IKBα mRNA levels were increased with a dietary choline level up to 1589.3 (*P* < 0.05), and then plateaued. In Figure 5, the mRNA abundances of JAK1, JAK2, STAT3b1, and STAT3b2 increased with choline addition to 1215.8, 1589.3, 1589.3, and 1589.3 mg/kg, respectively (*P* < 0.05), and then plateaued. Tyk2 mRNA levels were highest in 407.4 mg/kg choline diet (*P* < 0.05). However, dietary choline had no influence on mRNA abundance of IKKα, JAK3, and STAT3a in skin of juvenile grass carp. As shown in Figure 6, the NF-κB p65 protein level in the nucleus decreased with choline supplements to 1215.8 mg/kg (*P* < 0.05) then plateaued. The IKBα, p-STAT3 Tyr705, and p-STAT3 Ser727 protein levels in the nucleus were elevated with dietary choline addition to 1589.5 mg/kg, respectively (*P* < 0.05).

## Discussion

This research used a growth experiment similar to our earlier study (3), which found choline improved fish growth performances of juvenile grass carp (3). Furthermore, fish growth performances were deeply related to the immune function of immune organs (28), Hence, we explored how choline impacts the skin immune function of fish in this study.

### Dietary Choline Increased the Content of Choline and Its Metabolites and Upregulated the Gene Expression of Choline Transporter in Fish Skin

Dietary choline was absorbed by guts and through blood travels to different tissues where it can be oxidized, phosphorylated, and acetylated and can produce corresponding metabolites, such as betaine, PC, and ACh. In our research, dietary choline could increase the content of betaine, PC, and choline in fish skin. Results show that the optimal choline increased the choline content and corresponding metabolite in fish skin. In mammal enterocytes, choline can be transported through transport systems (11), such as choline transporter-like proteins (CTLs) and high-affinity choline transporter (CHT1) (11). In this research, we found that optimal dietary choline upregulated the mRNA levels of CTL2, CTL5, and CHT1, which indicated that skin absorbed choline through choline transporters CTL2, CTL5, and CHT1. Based on choline content and choline transporter mRNA abundance, the positive correlation is found in Table 5; we conjectured that the upregulation of those choline transporters' mRNA abundance might be related to the increase of choline contents in fish skin.

**Table 5:** Correlation analysis of parameters in the skin of juvenile grass carp (*Ctenopharyngodon idella*).

**Independent**	**Dependent**	**Correlation**	***P***
**parameters**	**parameters**	**coefficients**	
Choline	CTL2	0.981	<0.01
	CTL5	0.959	<0.01
	CHT1	0.927	<0.01
NF-kB P65	IL-1β	0.986	<0.01
	IFN-γ2	0.981	<0.01
	TNF-a	0.91	0.012
	IL-6	0.932	<0.01
	IL-12P40	0.982	<0.01
	IL-15	0.86	0.028
NF-kB P52	IL-1β	0.994	<0.01
	IFN-γ2	0.991	<0.01
	TNF-a	0.956	<0.01
	IL-6	0.952	<0.01
	IL-12P40	0.996	<0.01
	IL-15	0.906	0.013
c-Rel	IL-1β	0.949	<0.01
	IFN-γ2	0.978	<0.01
	TNF-a	0.919	<0.01
	IL-6	0.969	<0.01
	IL-12P40	0.984	<0.01
	IL-15	0.874	0.023
IkBa	NF-kB P65	−0.92	<0.01
	NF-kB P52	−0.94	<0.01
	c-Rel	−0.977	<0.01
	IKKβ	−875	0.022
	IKKγ	−899	0.015
STAT3b1	TGF-β1	0.944	<0.01
	IL-10	0.983	<0.01
	IL-11	0.96	<0.01
	IL-4/13A	0.953	<0.01
	IL-4/13B	0.981	<0.01
STAT3b2	TGF-β1	0.829	0.042
	IL-10	0.95	<0.01
	IL-11	0.879	0.021
	IL-4/13A	0.96	<0.01
	IL-4/13B	0.936	<0.01
JAK1	STAT3b1	0.937	<0.01
	STAT3b2	0.966	<0.01
JAK2	STAT3b1	0.92	<0.01
	STAT3b2	0.968	<0.01

Strikingly, choline has no impact on fish skin CTL4 mRNA abundance, which might be associated with ACh. CTL4 is involved in the synthesis of ACh in animals (37). However, in our research, choline did not influence the ACh content of grass carp skin, which provides an important basis for our hypothesis.

### Dietary Choline Reduced Skin Lesion Morbidity and Enhanced Immune Function in Fish Skin

*A. hydrophila* is a potentially pathogenic bacterium that can cause fish skin lesion and even lead to death (28). Hence, at the end of the growth test using *A. hydrophila* trail to study the skin lesion degree. During challenge with *A. hydrophila*, we found that dietary choline deficiency led to peak skin lesion morbidity (28.17%), whereas sufficient choline abundance decreased skin lesion morbidity (8.45%) in grass carp. Furthermore, the skin lesion resistance partially relies on skin immune response in fish (38). These results declared that sufficient dietary choline reduced skin lesion morbidity. In fish, the immune function was mainly influenced by antimicrobial peptides such as LEAP-2 and hepcidin and humoral components like C4 and IgM (39). The above results indicated that optimal dietary choline enhanced the ACP and LZ activities and C3, IgM, and C4 contents and increased mucin2, β-defensin LEAP-2B, and hepcidin mRNA abundance in fish skin. These results investigated that choline addition heightened the immune function in fish. The concentration of C4 in the optimal choline supplementation group was 3.39-fold that of the 142.2-mg/kg choline group, which was significantly higher than other enzyme activities and C3 content (1.36–1.89-fold). This indicates that the regulation of C4 by choline is more active and effective.

Interestingly, considering the differences between the vitamins mentioned in the introduction, we compared choline with VB7 and α-lipoic acid (20, 40). Unlike two other vitamins, choline deficiency did not affect the LEAP-2A mRNA level, which might be partly relevant to IKKα. IKKα improved the IL-22 level in mice (41), which enhanced LEAP-2A mRNA abundance in rainbow trout splenocytes (42). However, in this research, choline supplementation did not affect IKKα mRNA abundance in fish skin, which might support our hypothesis. Moreover, these data also illustrated that LEAP-2A might be more highly conscious by VB7 and α-lipoic acid in fish skin than dietary choline.

### Dietary Choline Enhanced Immune Function Referring to the mRNA Abundance of Inflammatory Factors in Fish Skin

Inflammation is a host defense mechanism, but overregulation of the inflammatory response disrupts the balance of immune function, leading to the deterioration of human diseases. Morimoto et al. observed that the inflammatory function of fish immune organs was affected by inflammatory cytokines (43). However, no research has studied the impacts of choline addition on inflammatory response in fish skin. In this research, we for the first time investigated that choline addition decreased the mRNA level of proinflammatory cytokines IFN-γ2, TNF-α, IL-15, IL-12p40, IL-6, and IL-1β and upregulation of the mRNA abundance of anti-inflammatory cytokines TGF-β1, IL-11, IL-10, and IL-4/13A in grass carp skin. In short, all of the above results illustrated that dietary choline supplement improved the immune function in fish skin.

Interestingly, the potential reasons for differential result are discussed as follows. First, our previous research found that an appropriate dietary VB7 could descend the mRNA abundance of IL-12p35 (rather than IL-12p40) (20). In contrast, in our study, appropriate choline increased fish skin IL-12p40 (not IL-12p35) mRNA abundance. This result might be related to TNF-α. In this research, appropriate dietary choline downregulated TNF-α mRNA levels in grass carp skin. In Atlantic salmon HK cells, TNF-α could increase the IL-12p40 (not IL-12p35) gene level (44), which supports our speculation. Moreover, these data also indicated that choline and VB7 had different regulatory mechanisms between different subtypes of the same gene (such as IL-12p35 and IL-12p40). Second, dietary α-lipoic acid and VB7 decreased the mRNA abundance of IL-17D, but dietary choline did not influence the IL-17D mRNA abundance in fish skin which might be relevant to tryptophan. Tryptophan catabolites could inhibit the production of IL-17 in mice (45). Previous studies have found that choline did not affect the content of tryptophan in the intestine (3), which might support our hypothesis and needs further investigation. In addition, these data also indicated that IL-17D is more easily regulated by dietary choline rather than α-lipoic acid and VB7 in fish skin. Third, choline insufficiency did not influence the TGF-β2 mRNA abundance in fish skin, which might be partly related to methionine. Methionine regeneration was mediated by choline in bovine neonatal hepatocytes (46). Methionine dipeptide supplementation has no impact on the TGF-β2 mRNA abundance of grass carp intestine (47), which needs further study. Moreover, previous studies found that dietary α-lipoic acid could upregulate the mRNA abundance of TGF-β2, indicating that TGF-β2 is more easily regulated by dietary α-lipoic acid rather than choline in fish skin.

### Dietary Choline Enhanced Immune Function Referring to JAK/STAT3 and NF-κB Signaling Molecules in the Fish Skin

A previous study found that inflammatory response is related to the mRNA expression of proinflammatory cytokines in fish (48). NF-κB p52, p65, and c-Rel are very important members of NF-κB signaling molecules which regulate the proinflammatory cytokines (49). NF-κB p65 and p52 were inhibited by IkBα in mammalian cells (50). Our data showed that the proinflammatory cytokine (IFN-γ2, IL1β, IL-6, TNF-α, IL-12p40, and IL-15) mRNA abundance was downregulated by optimal dietary choline in grass carp skin, implying that optimal dietary choline enhanced inflammatory function in fish skin. Furthermore, optimal dietary choline restrained the NF-κB signaling pathway by activating the IKBα protein level and decreasing the nuclear NFκBp65 protein level, as well as C-Rel, IKKβ, NF-κBp65, IKKγ, and NF-κBp52 (not IKKα) mRNA abundance in grass carp skin. C-Rel, NF-κB p52, and NF-κBp65 mRNA abundance positively correlated the pro-inflammatory cytokine mRNA abundance, shown in Table 5. At the same time, c-Rel, NF-κB p52, and NF-κB p65 mRNA abundance was positively related with IKKβ and IKKγ mRNA abundance, which was inversely correlated with IκBα mRNA abundance. These data elucidated that the optimal dietary choline downregulated the proinflammatory cytokine mRNA abundance which might be relevant to IKKβ and IKKγ/IκBα/NF-κB signaling in fish skin. Our results show that choline affects both canonical and non-canonical NF-κB signaling. However, there are few studies about the effect of choline on the NF-κB signaling pathway and only focus on canonical NF-κB. We speculate that cholesterol may be involved. Won et al. (51) found that plasma cholesterol was significantly influenced by the graded choline levels in juvenile olive flounder (*Paralichthys olivaceus*) (51). Optimal cholesterol inhibited both canonical and non-canonical NF-kappaB pathways in grass carp kidney and spleen (6), which might support our hypothesis and needs further investigation.

The STAT3 signaling pathway plays an important role in promoting the gene expressions of anti-inflammatory cytokines in humans (9). STAT3 (phosphorylation at tyrosine 705, Tyr705, and/or serine 727, Ser727) is also a member of the STAT family, which can be activated by the upstream signaling molecule Janus kinases (e.g., JAK2) (52). In this research, we for the first time investigated that choline addition increased the mRNA abundance of STAT3b1, STAT3b2, JAK1, and JAK2, as well as p-STAT3 Tyr705 and p-STAT3 Ser727 protein levels in fish skin (Figure 5), which implies that dietary choline upregulated most of anti-inflammatory cytokines which might be partly related to the STAT3 signaling pathway. Correlation analyses (Table 5) showed that anti-inflammatory cytokines (IL-10, TGF-β1, IL-4/13A, IL-11, and IL-4/13B) were positively correlated with the STAT3b1 and STAT3b2 mRNA abundance in fish skin. Our data showed that the weakened effect of the anti-inflammatory cytokine by dietary choline might be relevant to JAK1 and JAK2/STAT3 signaling in grass carp skin.

Interestingly, choline has no impact on IKKα mRNA abundance of grass carp skin, which may be relevant to IFN-γ. This research indicated that dietary choline deficiency increased fish skin IFN-γ mRNA abundance. In U937 cells, IFN-γ enhanced the protein kinase Cζ level (53), which increased IKKβ and IKKγ (not IKKα) gene abundance in Kupffer cells (54) Further research is needed to confirm this hypothesis. Similar phenomena were discovered in VB7, and α-lipoic acid from our previous research in grass carp skin, which may illustrate that vitamin B is not sensitive enough to IKKα in skin. Moreover, in human myeloid cells, the macrophage colony-stimulating factor (GM-CSF) could activate STAT3b (rather than STAT3a) (55). However, choline supplementation had no effect on GM-CSF concentrations in rat placenta (56). Therefore, we speculated that choline deficiency downregulated STAT3b1/b2 rather than STAT3a gene expressions in fish skin which may be caused by the decrease of GM-CSF. Further research is needed to confirm this hypothesis.

### Choline Requirements Based on Immune Indices

**Figure 7:**
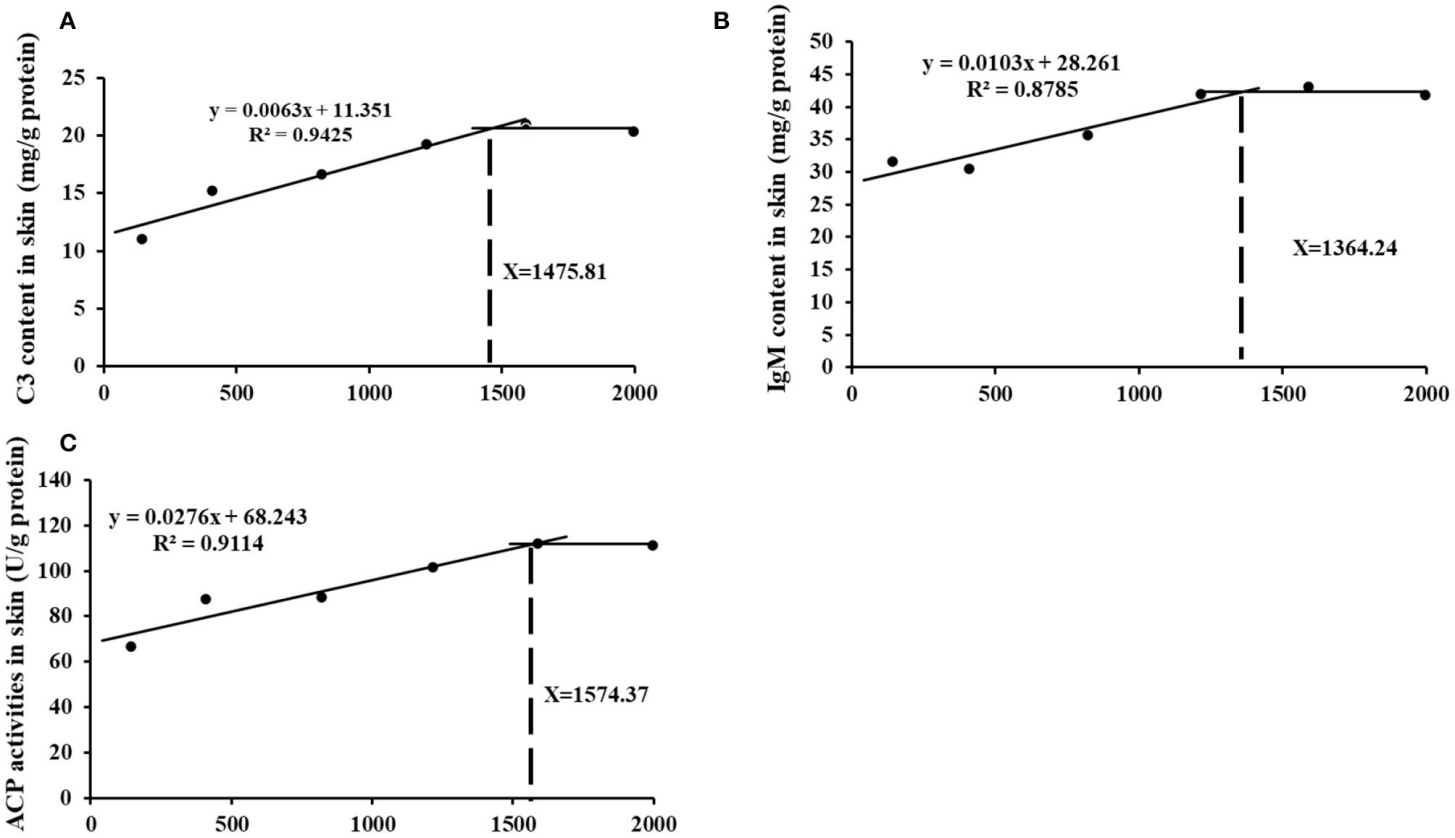
Broken-line analysis of C3 **(A)**, IgM **(B)**, and ACP content **(C)** in skin for grass carp (*Ctenopharyngodon idella*) fed diets containing graded levels of choline after infection with *A. hydrophila*.

The appropriate choline group advanced most of inflammatory indicators and receded skin lesion. Further, based on the skin immune indices (C3 and IgM contents as well as ACP activities) the choline requirements for grass carp (9.28–108.97 g) were estimated to be 1475.81, 1364.24, and 1574.37 mg/kg diet, respectively (As exhibited in Figure 7). The requirements for the majority of the skin immune index were higher than those for the growth (feed efficiency 1283.4 mg/kg diet) (3). Similar results were found in other vitamins like VB7 and VC in grass carp (19, 20). This result may illustrate that more choline or metabolites was needed for fish to resistance to bacterial infection.

## Conclusions

In summary, we reveal four primary, innovative, and interesting results. The results exhibited that dietary choline (1) could increase the contents of choline and its metabolites as well as choline transporter expression in the skin; (2) reduced the skin lesion and increased the contents of C3, IgM, and C4 and the activities of ACP and LZ, advanced mucin2, LEAP-2B, hepcidin, and β-defensin mRNA abundance, upregulated the mRNA abundance of IL-1β, IFN-γ2, TNF-α, IL-6, IL-12P40, and IL-15 in skin of juvenile grass carp, and downregulated the mRNA abundance of TGF-β1, IL-4/13A, IL-4/13B IL-11, and IL-10 in grass carp skin showed that choline enhanced the immune function of the skin; and (3) downregulated the mRNA abundance of IKKγ, IKKβ, c-Rel, NF-κBp65, and NF-κBp52 as well as protein level of NF-κBp65 in the nucleus, upregulated the mRNA and protein level of IkBα, and advanced the mRNA abundance of STAT3b1, STAT3b2, JAK1, and JAK2 as well as p-STAT3 Tyr705 and p-STAT3 Ser727 protein level in grass carp skin, indicating that choline-protected immune function might relate to the JAK/STAT3 and NF-κB signaling pathway in fish skin. In conclusion, choline enhanced the skin immune function relevant to JAK 1, 2/STAT3, and NF-κB signaling molecules in fish. Furthermore, based on the skin immune indices (C3 and IgM contents as well as ACP activities), the choline requirements for grass carp (9.28–108.97 g) were estimated to be 1475.81, 1364.24, and 1574.37 mg/kg diet, respectively.

## Data Availability Statement

The datasets presented in this study can be found in online repositories. The names of the repository/repositories and accession number(s) can be found in the article/supplementary material.

## Ethics Statement

The animal study was reviewed and approved by Institutional Animal Care and Use Committee of Sichuan Agricultural University.

## Author Contributions

Z-HY performed formal analysis, investigation, and writing—original draft. LF performed conceptualization, methodology, validation, data curation, and project administration. W-dJ performed data curation, validation, project administration, and writing—review & editing. PW performed conceptualization, funding acquisition, and resources. YL performed project administration. S-yK and LT performed resources. X-qZ performed conceptualization, methodology, supervision, funding acquisition, and supervision. All authors contributed to the article and approved the submitted version.

## Conflict of Interest

The authors declare that the research was conducted in the absence of any commercial or financial relationships that could be construed as a potential conflict of interest.
